# Depression among Patients with HIV/AIDS: Research Development and Effective Interventions (GAP_RESEARCH_)

**DOI:** 10.3390/ijerph16101772

**Published:** 2019-05-19

**Authors:** Bach Xuan Tran, Roger C. M. Ho, Cyrus S. H. Ho, Carl A. Latkin, Hai Thanh Phan, Giang Hai Ha, Giang Thu Vu, Jiangbo Ying, Melvyn W. B. Zhang

**Affiliations:** 1 Institute for Preventive Medicine and Public Health, Hanoi Medical University, Hanoi 100000, Vietnam; 2 Bloomberg School of Public Health, Johns Hopkins University, Baltimore, MD 21218, USA; carl.latkin@jhu.edu; 3 Department of Psychological Medicine, Yong Loo Lin School of Medicine, National University of Singapore, Singapore 119078, Singapore; pcmrhcm@nus.edu.sg; 4 Center of Excellence in Behavioral Medicine, Nguyen Tat Thanh University, Ho Chi Minh City 700000, Vietnam; 5 Department of Psychological Medicine, National University Hospital, Singapore 119074, Singapore; cyrushosh@gmail.com; 6 Institute for Global Health Innovations, Duy Tan University, Da Nang 550000, Vietnam; haipt.ighi@gmail.com (H.T.P.); giang.ighi@gmail.com (G.H.H.); 7 Center of Excellence in Evidence-based Medicine, Nguyen Tat Thanh University, Ho Chi Minh City 700000, Vietnam; giang.coentt@gmail.com; 8 Family Medicine & Primary Care, Lee Kong Chian School of Medicine, Nanyang Technological University, Singapore 639815, Singapore; yingjiangbo@gmail.com; 9 National Psychiatry Residency Program, Singapore 308440, Singapore; melvynzhangweibin@gmail.com

**Keywords:** HIV, AIDS, depression, scientometrics, bibliometric

## Abstract

Depression in people living with HIV (PLWH) has become an urgent issue and has attracted the attention of both physicians and epidemiologists. Currently, 39% of HIV patients are reported to suffer from depression. This population is more likely to experience worsening disease states and, thus, poorer health outcomes. In this study, we analyzed research growth and current understandings of depression among HIV-infected individuals. The number of papers and their impacts have been considerably grown in recent years, and a total of 4872 publications published from 1990–2017 were retrieved from the Web of Science database. Research landscapes related to this research field include risk behaviors and attributable causes of depression in HIV population, effects of depression on health outcomes of PLWH, and interventions and health services for these particular subjects. We identified a lack of empirical studies in countries where PLWH face a high risk of depression, and a modest level of interest in biomedical research. By demonstrating these research patterns, highlighting the research gaps and putting forward implications, this study provides a basis for future studies and interventions in addressing the critical issue of HIV epidemics.

## 1. Introduction

HIV/AIDS has become one of the major global health burdens. By the end of 2017, there were 36.9 million people living with HIV (PLWH) worldwide with 1.8 million new infection cases and 940,000 deaths [1]. Among them, 39% were reported to have suffered from depression [2]. Depression is a mental health disorder that is highly prevalent, and characterized by low mood, diminished self-worth, pessimistic thoughts, poor concentration, and biological symptoms (that of poor appetite and sleep difficulties) and increased withdrawal from social activities. Along with anxiety disorder, these psychiatric problems may result in chronic detrimental impairments and could even lead to suicidal ideation [3]. According to the recent World Health Organization (WHO) report, there have been over 300 million people living with depression and nearly 800,000 patients died due to suicide each year [4]. Depressive disorders have caused over 50 million years lived with disability (YLD) worldwide, accounting for 7.5% of global total YLD and, thus, are regarded as the single largest contributor to non-fatal health loss [5].

In individuals living with HIV, depression may worsen existing disease states and lead to poorer health outcomes. Prior research has revealed that depression is not only associated with higher HIV viral loads and lower CD4 cells count but also hastens the progression to AIDS and elevates the risk of mortality [6,7]. Furthermore, depression has been reported to reduce adherence to antiretroviral therapy (ART), weaken its therapeutic effects, and compromises the medication outcomes at both individual and population scale [8,9]. Adherence has been universally defined as “the extent to which a person’s behavior—taking medication, following a diet, and or executing lifestyle changes—corresponds with the agreed recommendations from a provider” [10]. As adherence to ART medications is instrumental in treatment effectiveness and clinical outcomes, ART interruption and discontinuation would worsen physical functioning, and along with depressive behaviors, this could result in further impairments in social relationships and a consequential reduced overall quality of life [11]. The comorbidity of HIV and depression typically results in longer onset depressive illnesses and more severe symptoms, such as higher distress and self-stigma, loss of appetite, and poorer sleep quality [12]. Additionally, the risk of experiencing moderate to severe depressive symptoms in patients that are non-adherent to ART was reported to be three-fold higher compared to adherent ones [13].

In recent years, there has been more research examining the effects of depression amongst individuals living with HIV. In 2014, a study by Arseniou et al. discussed methodology limitations and suggested further directions for diagnosis and management for depression in HIV-infected patients [14]. Maria G. Nani and colleagues reviewed the epidemiological characteristics and achievements on diagnoses and treatments for people suffering from both HIV and depression [15]. Meanwhile, depression among HIV and HCV co-infected patients was highlighted in a systematic review and meta-analysis by Renata Fialho et al. [16]. Nonetheless, to the best of our knowledge, there is currently no study performed a thorough bibliometric analysis that quantitatively and qualitatively examines the extant literature of depression in HIV population.

In order to demonstrate the global research trends as well as identify the research gaps of depression among HIV-positive people, we applied bibliometric analysis, which objectively evaluates the productivity of global researchers or institutions in this field [17]. Additionally, this study also aimed at reporting the trend of published articles over time and measured international growth based on databases of published literature. By pointing out the current trends. as well as presenting visual collaborating network of the studies on this research topic, we can effectively examine the development, productivity, and effective interventions of depression for HIV population and, thus, better inform researchers and physicians worldwide.

## 2. Materials and Methods 

### 2.1. Search Strategy

We chose the Web of Science as the online database to design a cross-sectional study for HIV/AIDS bibliography analyzing. The search query consisted of: HIV; human-immunodeficiency-virus, AIDS, and Acquired Immune Deficiency Syndrome (full strategy in Table S1). Document types rather than research articles and research reviews were excluded from the analysis. The language of the publications was restricted to English and only papers published before and in 2017 were chosen. 

### 2.2. Data Extraction

Data, including authors information (name and affiliation), the title of papers, the name of journal, keywords, and abstracts, were sorted by total citations and downloaded from the Web of Science. Citation reports automatically created by the Web of Science were also downloaded. All of the downloaded data were converted to xlsm form (Microsoft Excel, version 16, Microsoft, Washington, WA, USA) for data checking error. Standardization was performed by two analysts to merge the different name abbreviations of an author. In particular, one researcher checked if the name of an author appeared in more than one form, in detail “De Clercq E” or “Declercq E”, by coincidence of the authors’ workplace and email as the basic normalized criteria (for instance, Katholieke Universiteit Leuven and Catholic University of Leuven are one university in Belgium), and a second researcher verified the data. The procedure was followed by the filter of all the downloaded data by eliminating articles that were: (1) unoriginal reviews and papers, (2) unrelated to HIV/AIDS, and (3) not in English. Due to the vast records related to this topic, four researchers were involved and worked dependently in data filtering. Two research teams worked independently to make sure that the results matched. One research team, based on the document types that had been shown by the Web of Science, the title, and keywords of the papers, excluded 120,187 papers. The other team applied the same process to verify the results. Any conflict was solved by discussion.

### 2.3. Data Analysis

We analyzed data based on the total number of authors, year of publication, category, most popular keywords and their co-occurrence, citations, usages, and abstracts. After downloading and extracting data, we applied Macro, a programming code run in the Excel environment to calculate a country citation, and intra- and inter-country collaboration. A network of countries sharing co-authorships, the author keyword co-occurrence network and countries network were created by VOSviewer (version 1.6.8, Leiden University, Leiden, Netherlands). As for content analysis of the abstracts, we applied exploratory factor analysis to identify research domains emerging from all content of the abstract; loadings of 0.4 [18]. Jaccard’s similarity index was utilized to identify research topics or terms most frequently co-occurring with each other [19]. 

## 3. Results

### 3.1. Number of Published Items and Publication Trend

**Figure 1:**
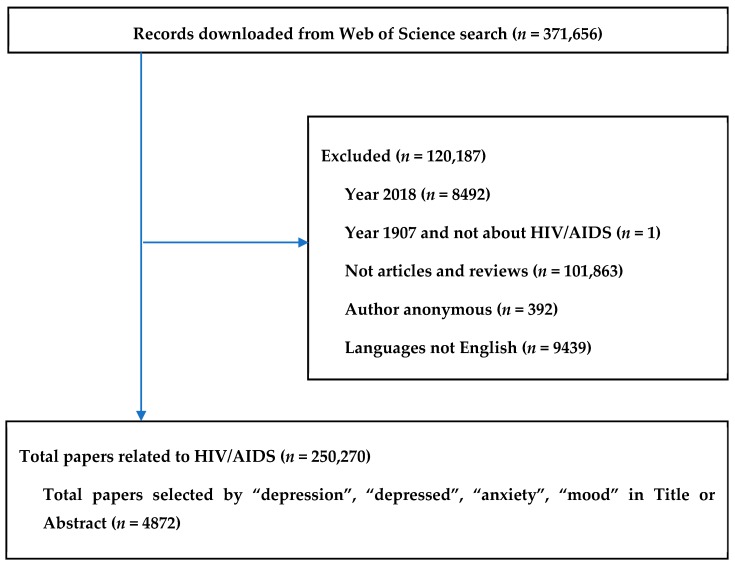
Selection of papers.

Figure 1 presents the paper selecting process, in which 250,270 papers relating to HIV/AIDS were identified. Among them, 4872 publications containing the terms “depression”, “depressed”, “anxiety”, and “mood” in either title or abstract were selected.

Not until the very first publication in 1990 did physicians start to pay more attention to depressive disorders in PLWH and significantly more papers were published, as illustrated by the considerable growth in the total number of papers over the research period. Especially in the last five years, total usage (the number of times being downloaded) and usage rates have increased highly. The papers published in 2006 have been particularly sought after, evidently by the substantial total citations (Table 1).

The number of articles counted by study settings is presented in Table 2. In total, there were 1791 cases in 60 countries. Being the epicenters of the world’s largest epidemic, it was not a surprise that the United States, South Africa, and Uganda were the most well-studied populations [20,21]. Many studies were also set up in such populous nations as China and India. Only a few study settings were located in such Asian developing countries as Vietnam, Taiwan, Thailand, etc.

### 3.2. Co-Authorship Network

**Figure 2:**
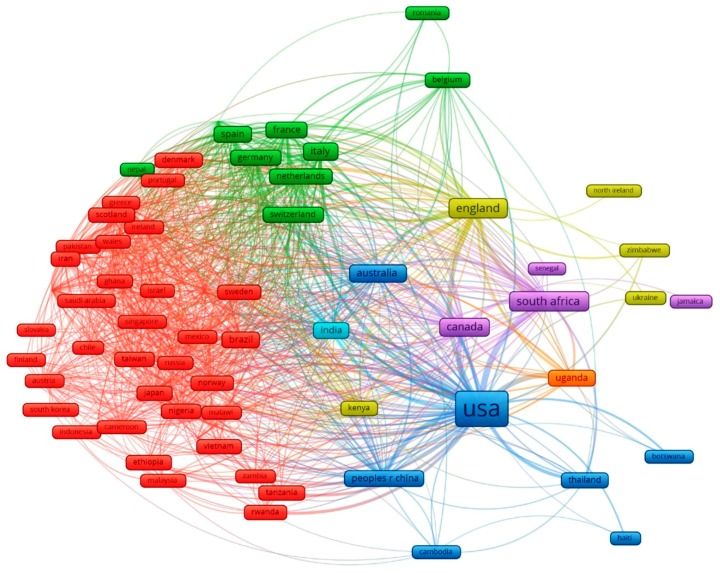
The global network among 68 countries having co-authorships of selected papers. The size of nodes shows the proportional contribution to the number of papers and the thickness of lines indicates the percentage of the number of collaborations.

Figure 2 illustrates the international collaboration network of 68 countries in depression research in HIV/AIDS. The United States of America appeared to be the knowledge hub of the world in this research topic, with the largest number of publications as well as densest co-authorships network, especially with its neighbor Canada and other large HIV populations, from South Africa, Uganda, to China, Thailand, and Cambodia. The collaborating networks were also based on geographical locations. The green cluster, for instance, indicates collaboration of Western European countries, including France, Germany, Switzerland, Netherlands, and Belgium. Meanwhile, the cluster in red revealed the cooperation of Asian nations (Singapore, Taiwan, Japan, South Korea, Vietnam, and Indonesia), the Middle East (Saudi Arabia, Israel, Iran), and Africa (Nigeria, Malawi, Ethiopia, Zambia, Tanzania, and Rwanda).

### 3.3. Keyword Co-Occurrence and Research Domains

**Figure 3:**
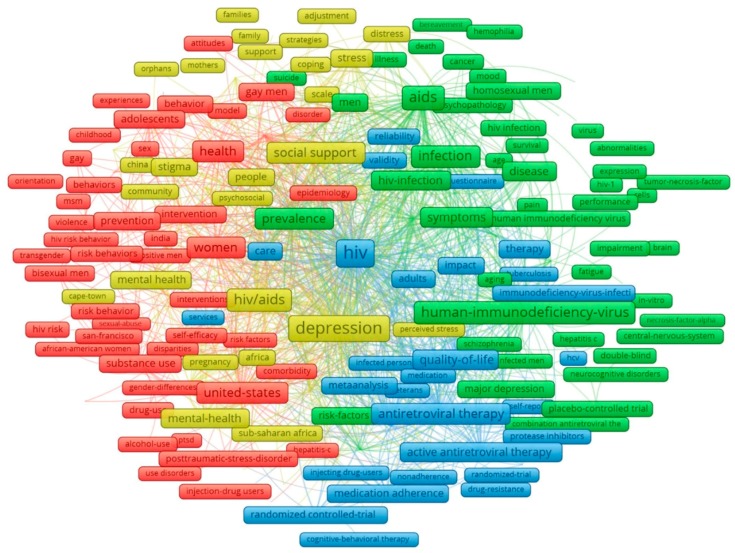
Co-occurrence of most frequent author’s keywords. Note: the colors of the nodes refer to principle components of data structure; the nodes size was scaled to the keywords’ occurrences; and the thickness of the lines was drawn based on the strength of the association between two keywords.

Analyses of keywords and abstract’s contents provided us with a better understanding of depressive disorders and related factors in PLWH. The principle components of keywords structure with the most frequent groups of terms are displayed in Figure 3. The clusters were emerged from 373 most frequent key words co-occurrence of at least 20 times. Nodes in red point out a number of HIV risk factors and behaviors (violence, sexual abuse, substance use) affecting vulnerable subjects, including women, adolescents, gay or bisexual men, transgender, and injection drug users, in highly infected populations, such as the United States and India. Green nodes focus on symptoms of HIV infection and associated mental diseases (major depression, schizophrenia, neurocognitive disorders), while the cluster in yellow characterizes major causes of these illnesses (stigma, distress), as well as efforts and strategies to ameliorate these problems (social and family support, community adjustment). The blue cluster refers to current treatments and healthcare services to improve patients’ quality of life.

As for the content analysis of abstracts by exploratory factor analysis, the top 50 emerging research domains are listed in Table 3. The most common domain came to unprotected sex behaviors, accounting for more than half of total cases. Depression in PLWH has also been explored through various aspects, including attributable causes (social stigma and discrimination, violence, substance abuse), affected issues and associated consequences (quality of life, medication adherence, suicidal ideation, and mortality), and possible interventions and coping strategies (medical care, and social support). Meanwhile, biomedical aspects, such as viral load and cell counts, and immune responses were not the choice of many researchers, as illustrated by the relatively low rank in the list (#25 and #35, respectively).

**Figure A1:**
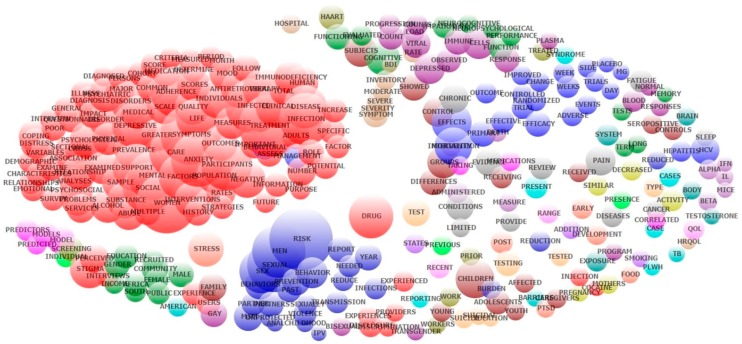
Co-occurrence of most frequent topics emerging from exploratory factor analysis of abstracts’ contents.

Exploratory factor analysis of abstracts’ contents also materialized the co-occurrence of the most frequent topics (Figure A1). Demographic and epidemiological characteristics of PLWH suffering from depressive symptoms and anxiety were grouped in the red cluster. Risk behaviors (sexual transmission, violence) and the interventions and preventions in different time courses (day, week(s), and year) were found to have strong correlation (blue nodes). Researchers also considered depressive responses in an immunological view (purple nodes), whereas such topics as neuropsychological performance, cognitive functioning, tests for symptoms and severity of depression, and specific patient groups did not show any apparent co-occurrence pattern with other terms or topics.

**Figure 4:**
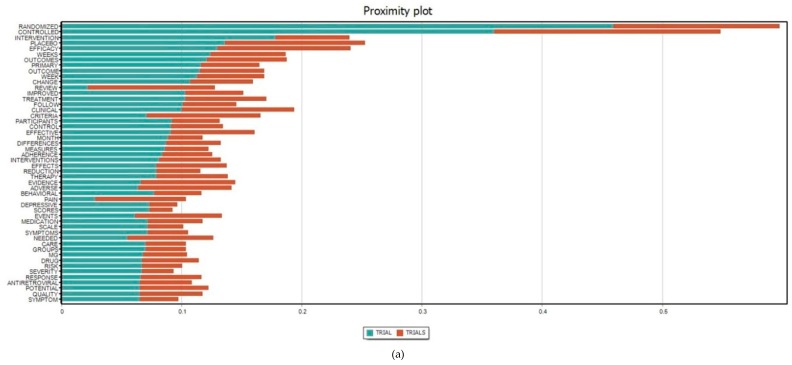

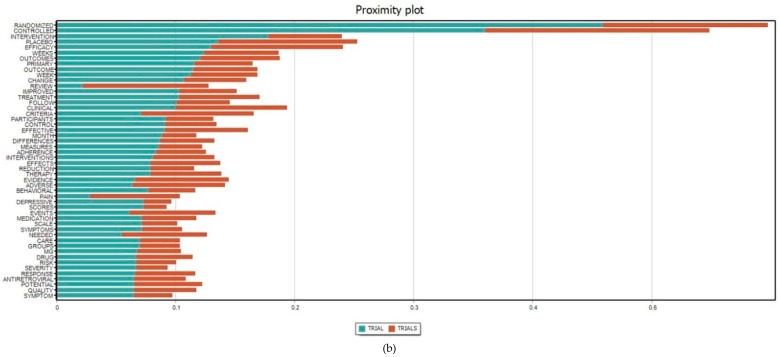
Proximity plots of the terms (**a**) “Intervention(s)” and (**b**) “Trial(s)” with the top 50 most frequent concurrence terms in all abstracts. The x-axis refers to the Jaccard coefficient that measures the similarity between finite sample sets and is defined as the size of the intersection divided by the size of the union of the sample sets.

Figure 4 presents the most frequent terms co-occurring with intervention(s) or trial(s) in the content analysis of all abstracts. The scope of interventions related to depression among HIV/AIDS covered behavioral, sexual, and treatment adherence interventions, at either individual or community levels, involving interrelationships and social supports, and measurements of various outcomes, including physical (e.g., pain), psychological, and depressive symptoms. Meanwhile, randomized controlled trial(s), which were applied to measure medication adherence and quality of potential therapy, was apparently the most common type of study setting.

## 4. Discussion

This study revealed the development of research on depression in PLWH in terms of quantity and current interests. Along with the significant increase of scientific literature volume, depression among HIV-infected patients has been extensively examined. Research landscapes related to this field include risk behaviors and attributable causes of depression in HIV population, effects of depression on health outcomes of PLWH, and interventions and health services for these particular subjects. Countries with large HIV populations, such as the United States of America, South Africa, Uganda, China, and India, possessed the highest number of empirical studies. Notable research gaps and future implications for further study on depression among PLWH are also discussed in this section.

In terms of collaborating framework, in addition to the regional collaboration, which is due to the similarities in socioeconomic characteristics, this research field has also witnessed the cooperation between the United States and other South East Asian nations, namely Thailand and Cambodia (Figure 2). By 2018, the Thai-U.S. partnership on HIV vaccine development has lasted for 25 years and the U.S. government has invested millions of US dollars in Thai research programs for HIV evaluation and optimization, as well as provided Thailand with service delivery and technical support through the President’s Emergency Plan for AIDS Relief (PEPFAR) since the fiscal year 2007 [22,23]. Meanwhile, Cambodia has received more than $150 million from USAID, which helped reduce the prevalence of adults with HIV and the HIV rate among sex workers to merely one third and provided roughly 80% of adult patients with adequate care and treatment [24]. With the help of the U.S., Thailand and Cambodia have earned international recognition as success stories in the fight against this global epidemic [24,25]. 

Risky sex behaviors, including unprotected sexual activities and anal sex, have been the most researched topic in the top 50 research domains (Table 3). Nevertheless, there were limited studies set up in Thailand—the home to populations exhibiting those risk behaviors most frequently, such as sex workers, transgender people, and homosexual men or men who have sex with men (MSM), to name a few (Table 2). Since female sex workers are usually exposed to a high risk of sexually transmitted diseases, work-related violence, and unwanted pregnancy, and experience both perceived and self-stigma due to their work characteristics, the prevalence of major depression of this group has been reported to be higher compared to general population [26,27,28,29]. Transgender people and MSM are also vulnerable to anxiety and depression, as they have to experience not only HIV-related discrimination and isolation but also prejudice against homosexuality [30,31]. Despite the fact that same-sex marriage has been allowed in many countries, homosexuality is still a controversial topic in Asia, and there is currently no Asian nation officially legalizing same-sex marriage [16,32]. Therefore, more study settings should be located in Asian developing countries, where the level of social judgement on the sex workers and LGBT community is relatively high, in order to demonstrate effective approaches to reach, test, and treat these key populations. 

Most of the existing interventions are focused on reduction of risk behaviors and social stigma, rather than interventions that target the infected populations (Figure 4). Since HIV is regarded as an incurable infection, it is essential to prevent incident HIV infections and the reduction of unprotected sex, injecting drugs, or substance abuse would lighten the global burden of HIV epidemic [33]. Additionally, stigma and discrimination, either perceived or self-stigma, is responsible for depressive symptoms in the majority of HIV-infected individuals [34]. Therefore, interventions addressing these problems would effectively lower the prevalence of HIV infection, in general, and depression among PLWH in particular. 

Although the field of depression in PLWH has been extensively studied, biomedical aspects of this research topic deserve more attention, since such research topics as viral load or immune responses currently hold relatively low positions (Table 3). In addition to psychological and somatic symptoms, biological factors also contribute to depression among HIV patients. A number of studies have recognized that chronic viral infections, including HIV, are able to affect immune system and influence the way the central nervous system mediates psychological status, resulting in neuropsychiatric consequences [35,36]. Biologically, HIV may trigger the release of inflammatory cytokines and induce sickness behaviors that are similar to depressive symptoms [37]. Additionally, while many antidepressants relieve the symptoms by elevating the level of a neurotransmitter called serotonin, HIV is capable of altering the precursor (tryptophan) and, thus, suppress the efficacy of the medications to a certain extent [38,39]. Evidences of neuronal damages have been recorded only a year after HIV infections and it been has reported that the use of antiviral therapies and the stage of the disease are associated with worsening depression [40,41,42]. 

There are several implications arising from the current study. Taking Thailand and Cambodia as the role models, developing countries could seek for investments from the U.S., considering that the US government not only offers various funds and organizations targeting HIV populations but also succeeded in supporting other countries to deal with the issue of depression among PLWH. Additionally, more studies should be set up in countries where the most-at-risk subjects commonly reside, such as Asian developing countries, in order to understand their nature, as well as the demographic characteristics and contextual factors, thus establishing more practical interventions. On the other hand, the biological correlation between HIV infection and depression requires more intensive research, which may make a great contribution to the diagnostic procedure and treatments for depression among individuals suffering from HIV. 

Even though we introduced a novel approach in summarizing and analyzing the extant literature, some limitations should be acknowledged. First, the involved databases were limited to only the Web of Sciences. Despite the fact that Web of Science contains the largest proportion of the literature of HIV/AIDS research, it is probably not fully representative of all data. Another limitation is that only publications in English were selected for this study. Additionally, the content analysis consisted solely of abstracts instead of full texts. Nevertheless, this modified bibliometric analysis puts forward a comprehensive overview of research trends as well as identifies current gaps in the literature of depression among PLWH.

## 5. Conclusions

In conclusion, by using bibliometric and scientometric analysis, this study presented the global research trends and interests, pointed out the research gaps of available publications, and suggested several implications for depression of HIV-positive individuals. In spite of the fact that this field has attracted a great deal of attention and been extensively studied, more efforts should be made to fulfill the lack of empirical study in developing countries and biomedical investigation on the correlation between HIV and depression.

## Figures and Tables

**Table 1:** General characteristics of publications.

Year Published	Total Number of Papers	Total Citations	Mean Cite Rate per Year	Total Usage Last 6 Month	Total Usage Last 5 Years	Mean Use Rate Last 6 Month	Mean Use Rate Last 5 Year
2017	428	565	1.32	663	2018	1.55	0.94
2016	378	1775	2.35	404	3683	1.07	1.95
2015	330	2811	2.84	272	3896	0.82	2.36
2014	329	3978	3.02	198	4083	0.60	2.48
2013	338	5078	3.00	187	4867	0.55	2.88
2012	323	8434	4.35	208	4390	0.64	2.72
2011	237	6059	3.65	123	2680	0.52	2.26
2010	248	6525	3.29	111	2141	0.45	1.73
2009	218	6315	3.22	102	1758	0.47	1.61
2008	193	7629	3.95	114	1825	0.59	1.89
2007	153	7145	4.25	107	1516	0.70	1.98
2006	181	10,994	5.06	130	2044	0.72	2.26
2005	147	7174	3.75	69	1135	0.47	1.54
2004	127	5453	3.07	53	666	0.42	1.05
2003	121	6364	3.51	64	945	0.53	1.56
2002	116	5739	3.09	46	605	0.40	1.04
2001	109	8811	4.75	49	968	0.45	1.78
2000	105	7415	3.92	63	1026	0.60	1.95
1999	85	4077	2.52	23	343	0.27	0.81
1998	109	3682	1.69	23	405	0.21	0.74
1997	108	8473	3.74	97	1114	0.90	2.06
1996	110	4912	2.03	24	288	0.22	0.52
1995	85	2706	1.38	13	217	0.15	0.51
1994	81	2850	1.47	9	128	0.11	0.32
1993	83	3940	1.90	13	202	0.16	0.49
1992	63	3548	2.17	12	149	0.19	0.47
1991	49	2302	1.74	5	71	0.10	0.29
1990	1	24	0.86	0	1	0.00	0.20

Mean cite rate per year = total citations/[total citations*(2018-that year)]. Total usage: total downloads. Mean use rate last 6 months = total usage last 6 months/total number of papers. Mean use rate last 5 years = total usage last 5 years/(total number of papers*5).

**Table 2:** Number of papers by countries as study settings.

#	Country Settings	Frequency	%	#	Country Settings	Frequency	%
1	United States	432	22.7%	31	Haiti	13	0.7%
2	South Africa	203	10.7%	32	Iran	13	0.7%
3	Uganda	102	5.4%	33	Hong Kong	10	0.5%
4	China	89	4.7%	34	Spain	10	0.5%
5	India	73	3.8%	35	Denmark	9	0.5%
6	Ireland	73	3.8%	36	Jersey	9	0.5%
7	Canada	62	3.3%	37	Dominica	8	0.4%
8	Brazil	46	2.4%	38	Dominican Republic	8	0.4%
9	Australia	43	2.3%	39	Georgia	8	0.4%
10	United Kingdom	41	2.2%	40	Netherlands	8	0.4%
11	Kenya	35	1.8%	41	Ukraine	8	0.4%
12	Viet Nam	32	1.7%	42	Chile	7	0.4%
13	Tanzania	28	1.5%	43	Germany	7	0.4%
14	Niger	27	1.4%	44	Jamaica	7	0.4%
15	Nigeria	27	1.4%	45	Lesotho	6	0.3%
16	Puerto Rico	24	1.3%	46	Malaysia	6	0.3%
17	Rwanda	24	1.3%	47	Peru	6	0.3%
18	Taiwan	22	1.2%	48	Sweden	6	0.3%
19	Ethiopia	21	1.1%	49	Colombia	5	0.3%
20	Mexico	21	1.1%	50	Ghana	5	0.3%
21	Thailand	20	1.1%	51	Mali	5	0.3%
22	Zambia	18	0.9%	52	Namibia	5	0.3%
23	Malawi	17	0.9%	53	Norway	5	0.3%
24	Zimbabwe	17	0.9%	54	Russian Federation	5	0.3%
25	Botswana	16	0.8%	55	Cambodia	4	0.2%
26	France	16	0.8%	56	Congo	4	0.2%
27	Japan	16	0.8%	57	Congo	4	0.2%
28	Nepal	15	0.8%	58	Israel	4	0.2%
29	Cameroon	14	0.7%	59	Senegal	4	0.2%
30	Italy	14	0.7%	60	Singapore	4	0.2%

**Table 3:** Top 50 research domains emerged from exploratory factor analysis of all abstracts’ contents.

No	Name	Keywords	Eigen Value	Freq	%cases
1	Sex Sexual; Unprotected	sex; unprotected; sexual; anal; partners; msm; risk; behaviors; prevention; behavior; men; partner; sexually	3.81	7005	54.74%
2	Psychological Distress; Emotional	distress; psychological; emotional; anxiety; physical	1.55	3364	45.28%
3	Medical Care	care; providers; medical; services; barriers; primary; interviews	1.81	3455	43.64%
4	Depressed	depressed; anxiety	1.30	2035	40.62%
5	Social Stigma	stigma; discrimination; social; support; disclosure; perceived; experiences	2.39	3374	39.16%
6	Psychiatric; Disorder	psychiatric; disorder; disorders; major; interview; diagnosis	2.89	3188	38.36%
7	Coping Strategies; Social Support	coping; strategies; support; social	1.41	2705	35.55%
8	Scale; Hospital	scale; hospital; questionnaire; total; score	1.31	2390	33.07%
9	Depressive	depressive; symptoms	1.29	2132	32.22%
10	Antiretroviral	antiretroviral; therapy; haart; adherence	2.66	2741	29.80%
11	Adherence To Medication	medication; adherence; medications; antiretroviral; taking	1.33	2384	29.02%
12	Cancer Pain; Chronic Diseases	cancer; pain; diseases; chronic; review; conditions	1.64	1930	28.08%
13	Mortality; Disease Progression	mortality; disease; progression; death	1.34	1680	26.89%
14	Provide Evidence	evidence; limited; review; trials; provide	1.39	1702	26.83%
15	Neuropsychological; Memory Performance	neuropsychological; performance; neurocognitive; impairment; memory; cognitive; tests; function; functioning	2.76	2292	25.92%
16	Violence (IPV); Partner	ipv; violence; partner; women; pregnancy	1.93	1640	24.63%
17	Disorder (PTSD)	ptsd; stress; post; disorder	1.70	1570	24.20%
18	Randomized; Controlled Trial	randomized; trial; controlled; placebo; trials; intervention; efficacy	3.30	2193	24.20%
19	Substance Abuse	abuse; substance; alcohol; childhood	1.61	1800	23.60%
20	Month Follow	month; follow; period; year	1.52	1579	23.19%
21	Control	controls; control; subjects; seropositive	1.51	1422	22.95%
22	Intervention Program	program; community; intervention	1.46	1266	22.48%
23	Examine	examine; purpose; relationships	1.30	1269	21.82%
24	Drug Users	users; drug; injection; cocaine	2.17	1457	20.79%
25	Viral Load; Cells Count	load; viral; count; counts; cells	1.92	1703	20.55%
26	Test	testing; test; tested; tests	1.42	1257	20.40%
27	Gay Men	gay; bisexual; men	1.88	1400	20.38%
28	Life Quality	quality; life; qol; hrqol	2.09	1619	20.32%
29	Human Immunodeficiency	immunodeficiency; human; syndrome	2.26	1891	20.30%
30	Side Effects	effects; side	1.43	1142	19.77%
31	Female	female; male; gender	1.54	1168	19.03%
32	Cross-Sectional	sectional; cross; survey	2.36	1703	18.80%
33	Symptom Severity	symptom; fatigue; sleep; severity; pain	1.59	1177	18.27%
34	Children; Family Caregivers	children; caregivers; affected; mothers; family	2.05	1241	18.00%
35	Response	responses; response; immune	1.25	976	16.07%
36	Persons	persons; states	1.28	756	14.57%
37	Infections; Sexually	infections; sexually; diseases	1.27	883	14.43%
38	Physical	physical	1.24	556	11.41%
39	Perceived	perceived; received	1.26	600	11.10%
40	Brain	brain; system	1.48	505	9.32%
41	Case	cases; case	1.32	491	9.20%
42	History	history	1.36	439	9.01%
43	Youth; Adolescents	youth; adolescents; young	1.57	514	8.50%
44	Adverse Events	events; adverse	1.39	488	8.48%
45	South Africa	africa; south	1.68	584	8.23%
46	Work	workers; work	1.35	401	7.45%
47	Long-Term	long; term	2.15	510	6.69%
48	Suicidal Ideation	suicidal; ideation; suicide	1.98	447	5.58%
49	Hepatitis C Virus (HCV)	hcv; hepatitis	1.79	360	5.25%
50	Testosterone	testosterone; body	1.37	238	4.52%

## Supplementary Materials

The following are available online at https://www.mdpi.com/1660-4601/16/10/1772/s1, Table S1: Search strategy on Web of Science.
