# Supplementary material for: Depression among Patients with HIV/AIDS: Research Development and Effective Interventions (GAPRESEARCH)
Source: Int J Environ Res Public Health. 2019 May 19;16(10):1772. doi: 10.3390/ijerph16101772 (PMC6571985; doi:10.3390/ijerph16101772)
Supplement: Supplementary file 1 [file ijerph-16-01772-s001.pdf]

Table S1. Search strategy on Web of Science

No	Search query	Result
1	TS=HIV	343,339
2	TS=(HIV AND AIDS)	96,659
3	TS=(human-immunodeficiency-virus)	120,344
4	TS=(Acquired-Immune-Deficiency-Syndrome)	4,852
5	#1 OR #2 OR #3 OR #4	371,656
